# Patient rights and consent form language about intraoperative audiovisual recording

**DOI:** 10.1007/s00464-025-12010-x

**Published:** 2025-07-17

**Authors:** Daniel R. S. Habib, Kavita Prasad, Marina Aweeda, George Lin, Anthony E. Bishay, Yue Gao, Dandan Liu, Alexander Langerman

**Affiliations:** 1 Vanderbilt University School of Medicine, Nashville, TN USA; 2 Department of Otolaryngology – Head and Neck Surgery, Vanderbilt University Medical Center, 1211 Medical Center Dr, Nashville, TN 37232 USA; 3 Michigan Medicine, Kellogg Eye Center, Ann Arbor, MI USA; 4 Department of Biostatistics, Vanderbilt University Medical Center, Nashville, TN USA; 5 Department of Radiology and Radiological Sciences, Vanderbilt University Medical Center, Nashville, TN USA; 6 Center for Biomedical Ethics and Society, Vanderbilt University Medical Center, Nashville, TN USA

**Keywords:** Clinical ethics, Consent form, Decision making, Informed consent, Intraoperative recording, Patient rights

## Abstract

**Background:**

Intraoperative audiovisual recordings offer benefits in quality improvement, education, and research, but challenges hinder their routine use. No comprehensive guidelines exist on how intraoperative recordings should be communicated to patients, and no previous study has examined how informed consent documents (ICDs) address this topic. This study aims to analyze procedural consent forms for themes and readability of disclosures related to intraoperative audiovisual recording.

**Methods:**

ICDs were collected from 104 high-volume U.S. hospitals identified via the American Hospital Association Annual Survey Database. Measures included hospital demographics (public/private, academic/non-academic, region, and Social Vulnerability Index), Flesch-Kincaid reading level, and audiovisual recording themes labeled by two independent researchers. Analyses examined the distribution of consent themes by hospital type and the association between reading level and the number of themes with U.S. census region, hospital type, and Social Vulnerability Index as covariates using ordinal logistic regressions.

**Results:**

Of 104 ICDs, 70 contained text about procedural recording. All 70 forms discussed modality, 66 (94.3%) discussed recording purpose, 38 (54.3%) discussed patient safeguards, and 10 (14.3%) discussed patient rights. The median reading level was 15.0 (IQR: 12.5–17.7), equivalent to third year of college. Higher reading level (aOR = 1.14, 95% CI 1.04–1.24) and academic hospital status (aOR = 2.83, 95% CI 1.11–7.23) were associated with more subthemes.

**Conclusion:**

Most ICDs addressed recording modality and purpose but inadequately covered safeguards and patient rights. The median reading level significantly exceeded the recommended sixth- to eighth-grade standard. These findings will help guide ICD development to include often overlooked themes and use accessible language about intraoperative recording.

**Supplementary Information:**

The online version contains supplementary material available at 10.1007/s00464-025-12010-x.

Audiovisual recording in operating rooms (ORs) is becoming more routine [1]. Ceiling-mounted, head-mounted, and operative light cameras are most commonly used for visual recording, while video cameras with audio capabilities, ceiling-mounted and surgeon-worn microphones are most commonly used for audio recording [2]. As technology advances and audiovisual recording becomes more feasible, intraoperative recording is increasingly being utilized for quality improvement, education, and research purposes [3–5]. However, this innovative integration of technology into the OR has raised important questions about legal and ethical considerations, patient and personnel privacy, and confidentiality [6, 7].

Despite facilitating knowledge-sharing, recording procedures have elicited variable feedback and policies from a range of stakeholders [8]. Professional societies (e.g., American Medical Association [AMA], Joint Commission on Accreditation of Healthcare Organizations) and hospital boards/trusts vary in procedural recording consent guidelines [9–12]. Additionally, OR staff often express concerns about recording anonymity [13], and many surgeons in training report that OR recording would change their behavior [14]. In the last decade, surgeons have begun sharing images and videos of their cases on the internet [15], bringing more attention to the ethics of intraoperative recording and garnering pushback from patients [16]. While some patients believe that intraoperative recording can enhance surgical training, quality, and safety as well as their own understanding of the procedure [15, 17], others expressed concerns about privacy and identifiability [8]. Prior studies have highlighted some of the risks [5] and benefits [18] of intraoperative recording, but consensus guidelines for its appropriate use have yet to be produced [8, 13, 14, 19].

Informed consent is vital for protecting patient autonomy and encouraging honest discussions between patients and their providers [20]. Prior work developing ethical recommendations for patients to consent to intraoperative recording suggests that the consent form (or informed consent document, ICD) should clearly state the recording's purpose, audience, accessibility, and storage duration [8]. Patients should also be given the right to refuse without impacting the quality of care they receive. Failure to address these aspects in the ICD may result in incongruence between the surgeon's interpretation of permission to use audiovisual media and the patient's intentions [8, 18, 21]. However, ICDs are frequently criticized for their poor readability [22] and use of complex language [23], potentially causing a discrepancy between the information provided to patients and their understanding of it. This prompts the question of how ICDs currently describe audiovisual recording and whether patients are receiving adequate information before consenting.

To date, no study has examined procedural ICD language regarding intraoperative recording. This work aims to analyze the reading level and content of procedural consent documents to determine what information hospitals currently include about intraoperative audiovisual recording and where ICD guidelines can be improved to allow for better patient understanding.

## Materials and methods

### Sample selection

This is a secondary analysis of a national sample of 104 procedural ICDs collected from 126 hospitals approached under a prior research study; the methodology has been published previously [24, 25]. Using the American Hospital Association (AHA) Annual Survey Database [26], general procedural (i.e., not procedure-specific or audiovisual-specific) ICDs were collected from the highest surgical volume U.S. private, public, academic, and non-academic adult hospitals from each state, with each hospital being allowed to meet multiple categories (e.g., the same hospital being both academic and private). If the first institution failed to respond in four contact attempts or declined participation, the next largest hospital of the same type in the same census division was contacted. Hospital demographic information (i.e., US county, academic or non-academic, and private or public) was collected. Moreover, the social vulnerability index (SVI) was determined from the Centers for Disease Control and Prevention (CDC) for each hospital’s location as a quantifiable metric for the impact of social determinants of health on study outcomes [27]; we hypothesize that because low socioeconomic status is associated with lower reading comprehension [28], ICDs of hospitals in low SVI areas may exhibit lower reading level tailored to their patient populations. Content analysis was conducted to identify the frequency of various disclosures in the ICD. This work generated the database used for the downstream analyses described below. This study was approved as exempt by the Vanderbilt Institutional Review Board (IRB #210716).

### Audiovisual disclosure analysis

For this study, disclosure clauses regarding audiovisual recording were extracted from our database. We analyzed the text from these clauses for Flesch-Kincaid reading level [24, 29–31], characters, words, and syllables per word. We also conducted a thematic analysis of the content of these disclosures. Two researchers (DH, KP) developed a preliminary codebook before independently identifying main themes and subthemes in the collection of disclosure clauses. We compared the independent thematic coding, resolved discrepancies with the senior author (AL), and generated a revised codebook. We then used this revised codebook to repeat the coding of these audiovisual disclosure clauses. The Standards for Reporting Qualitative Research (SRQR) checklist [32] is also presented in Supplement 1.

### Statistical analysis

Descriptive statistics were performed for hospital demographics and ICD characteristics. Themes and subthemes were summarized by counts and percentages. Text characteristics were summarized by mean, standard deviation, and median. Ordinal logistic regressions were performed to assess the effect of reading level on the number of themes and number of subthemes with U.S. census region, hospital type (public/private and academic/non-academic), and SVI as covariates. Logistic regressions were fitted for specific subthemes by reading level, hospital type, and SVI. Linear regression was performed to assess the effects of hospital type, region, and SVI on reading level. For ordinal logistic and logistic regressions, odds ratios (ORs) and 95% confidence intervals (CIs) were reported. For the linear regression, estimates and 95% CIs were reported. All statistical analyses were conducted using R (version 4.4.1).

## Results

Of 104 general procedure ICDs collected in the primary study [24], 70 (67%) contained text about procedural recording. The median word count of audiovisual recording text was 35.5 (interquartile range [IQR]: 27.0–61.0), and the median reading level of audiovisual recording text was 15.0 (IQR: 12.5–17.7), equivalent to third year of college. Table 1 shows audiovisual text examples with varying reading levels.

**Table 1:** Examples of audiovisual text at varying reading levels

Flesch-Kincaid reading level	Examples of informed consent audiovisual disclosure
8.7	I understand the staff may take pictures and videos during my procedure. I agree to this as long as my face and name are not used. I understand that every attempt will be made to protect my identity. I understand that some of these photographs/videotapes may be used for teaching and may not be maintained or be a part of my medical record. I also understand that photographs/videotapes to plan, monitor, or document my treatment may be part of my medical record
15.7	I understand that the provider may need to take photographs, video, and audio recordings to document a medical condition, help with diagnosis and treatment, and assist with the procedure. l also understand that the images of all or part of my procedure may be recorded or others may be in the room to observe for educational, research, quality or other healthcare purposes may be used for with patient identifiers removed
21.6	I consent to the photographing or videotaping of the operation to be performed, including appropriate portions of my body, for medical, educational, and medical record documentation purposes, provided said photographs and videotapes are maintained and released in accordance with medical record regulations. I consent to the admittance of observers to the use of closed-circuit television and the taking of photographs, including motion pictures, provided that my name is not used in this connection

Examples are comprised of combinations of sentences from multiple informed consent documents to retain the anonymity of the hospitals and are provided for example purposes only

Of the 70 ICDs with audiovisual recording text, four major themes were identified: modality, purpose, patient rights, and safeguards (Table 2). Modality encompasses the method by which media was obtained and stored. The most common modality included in audiovisual text was photography (N = 70, 100%), followed by video (N = 59, 84.3%), closed-circuit televising (N = 12, 17.1%), graphics (N = 7, 10%), and audio (N = 6, 8.6%).

**Table 2:** Number and percent of procedural informed consent documents containing intraoperative recording themes

Themes	Number (Percent) of ICDs
Modality	70 (100.0%)
Photo	70 (100.0%)
Video	59 (84.3%)
Closed-circuit televising	12 (17.1%)
Graphics	7 (10.0%)
Audio	6 (8.6%)
Purpose	66 (94.3%)
Education	54 (77.1%)
Research	31 (44.3%)
Documentation	20 (28.6%)
Care	17 (24.3%)
Medical not otherwise specified	16 (22.9%)
Quality	16 (22.9%)
Observers	7 (10.0%)
Reimbursement	2 (2.9%)
Marketing	2 (2.9%)
Social media	1 (1.4%)
Safeguards	38 (54.3%)
Deidentification	30 (42.9%)
Recording only appropriate body parts	6 (8.6%)
Abiding by applicable laws	4 (5.7%)
Confidentiality	3 (4.3%)
Disposal	1 (1.4%)
Storage	1 (1.4%)
Patient rights	10 (14.3%)
Additional purpose	5 (7.1%)
Consent withdrawal	3 (4.3%)
Refusing recording use for a specific purpose	2 (2.9%)
Ownership	2 (2.9%)
Access	1 (1.4%)
Approval	1 (1.4%)
Compensation	1 (2.9%)

*ICD* informed consent document

Sixty-six (94.3%) clauses contained information on the purpose of recording. The most common purpose was education (N = 54, 77.1%), followed by research (N = 31, 44.3%), documentation (N = 20, 28.6%), care (N = 17, 24.3%), “medical” not otherwise specified (N = 16, 22.9%), quality improvement (N = 16, 22.9%), observation (N = 7, 10%), reimbursement (N = 2, 2.9%), marketing (N = 2, 2.9%), and social media (N = 1, 1.4%).

Thirty-eight (54.3%) clauses contained information about patient safeguards. The most common safeguard by far was deidentification (N = 30, 42.9%), followed by recording only appropriate body parts (N = 6, 8.6%), abiding by applicable laws (N = 4, 5.7%), maintaining confidentiality (N = 3, 4.3%), media disposal (N = 1, 1.4%), and media storage (N = 1, 1.4%).

Only 10 (14.3%) clauses discussed patient rights, including re-contact for using media for an additional purpose (N = 5, 7.1%), consent withdrawal (N = 3, 4.3%), refusing recording use for a specific purpose (N = 2, 2.9%), and ability to access recording (N = 1, 1.4%). Forms also discussed waiving patient rights to compensation (N = 1, 1.4%), final product approval (N = 1, 1.4%), and ownership (N = 2, 2.9%).

Neither reading level (aOR = 1.003, 95% CI 0.91–1.10) nor being an academic hospital (aOR = 1.55, 95% CI 0.58–4.16) has a significant impact on the number of main themes. However, higher reading level (aOR = 1.14, 95% CI 1.04–1.24; Table 3) and being an academic hospital (aOR = 2.83, 95% CI 1.11–7.23) are associated with a higher number of subthemes, suggesting more thorough discussion of given themes when present.

**Table 3:** Multivariable ordinal logistic regression of number of subthemes by reading level, hospital type, region, and social vulnerability index

Variable	aOR (95% CI)	P-value
**Reading Level**	**1.14 (1.04–1.24)**	**.007**
**Academic (vs Non-academic)**	**2.83 (1.11–7.23)**	**.034**
Public (vs Private)	1.29 (0.53–3.14)	.573
Midwest (vs Northeast)	0.29 (0.08–1.10)	.074
South (vs Northeast)	0.33 (0.08–1.33)	.125
West (vs Northeast)	0.27 (0.06–1.15)	.083
Social Vulnerability Index	1.62 (0.20–13.00)	.652

*aOR* adjusted odds ratio; *CI* confidence interval; Significant findings (p < 0.05) are bolded

Since reading level is associated with higher number of subthemes, we wanted to explore if hospital demographics affected reading level. Although reading level was not significantly associated with hospital type and region, reading level was expected to increase by 7.03 with each unit of increase in SVI (95% CI 1.47–12.60).

Since academic hospitals have a higher likelihood of including more subthemes even after controlling for reading level, we hypothesized that certain subthemes (deidentification, education, additional observers, and research) would more likely be included in ICDs of academic hospitals. However, academic hospital ICDs are not associated with significantly higher odds of discussing deidentification (aOR = 1.67, 95% CI 0.58–4.81), education (aOR = 2.95, 95% CI 0.71–12.29), additional observers (aOR = 2.18, 95% CI 0.37–12.78), or research (aOR = 1.73, 95% CI 0.57–5.22). See Supplement 2 for more details.

## Discussion

In our analysis of disclosure clauses regarding intraoperative photo and video recording in procedural ICDs, we found that two-thirds of the ICDs analyzed contained text about procedure recording. Of these 70 ICDs, all contained language about the modality of recording, and nearly all documents explained the purpose behind the recordings. This contrasts sharply with the lack of language around safeguards and patient rights in most ICDs of the sample. Only about half of ICDs with audiovisual disclosures contained language about patient safeguards, and less than 15% discussed patient rights. Hospitals must close the gap by increasing discussion of safeguards and patient rights regarding intraoperative audiovisual recording. As visual documentation in the OR continues to expand, the number of ICDs containing text about such protocols will likely need to increase accordingly.

There was a wide range in the number of subthemes included, perhaps reflecting hospitals’ efforts to navigate this topic in the absence of comprehensive ICD guidelines on how audiovisual recording should be used, stored, and presented to patients [8]. For instance, the American Medical Association (AMA) addresses informed consent, confidentiality, and security risks of intraoperative recording for education but falls short in discussing access rights and identification risk reduction [8]. The American College of Surgeons’ outline for ICDs does not discuss any aspect of audiovisual recording [19].

Reading level is also a key consideration in ICD creation. The average reading level was much higher than the recommended sixth- to eighth-grade reading level for ICDs [33]. The high standard deviation for average reading level and word count speaks to the high variability in the text dedicated to audiovisual recording within consent documents. Text with too high of a reading level might not be understood well enough by the patient to be useful, but text with too low of a reading level might not provide adequate explanation to the patient. Despite having no significant effect on the number of main themes, a higher reading level was associated with slightly higher odds of the ICD including more subthemes. Surprisingly, higher SVI was associated with higher ICD reading level. However, the large confidence interval speaks to the potential limited applicability of using the model as well as the large variation in SVI. More in-depth analysis to understand what other factors affect this relationship is needed. Overall, the goal should be to maintain the lowest reading level that allows the ICD to still adequately explain important topics; patient-centered work on consent language is warranted.

### Notable gaps in informed consent documents

Of the seventy forms, only one discussed social media. Given the prevalence of procedural recordings posted on social media, more work is needed to provide patients with information regarding where recordings could be disseminated. This is essential since media posted online can be instantaneously shared with millions of people and is permanent, thus putting into question whether the right to rescind holds merit in this case. Patients’ decisions to consent may change if these consequences are explained in full and thus require more attention. As audiovisual recording becomes more commonplace, so does the availability of content that might be posted on social media as well as the need to inform patients about this possibility.

Importantly, only two ICDs discussed hospital ownership of the media product. In one form, the patient could request access to a copy. The other form discussed consent for audiovisual recording while waiving all rights to the audiovisual recordings, including final product approval and compensation until the patient withdraws their consent in writing. Establishing written documentation and agreeing to such terms is critical because ownership topics (e.g., the right to access, edit, destroy, and sell) are all without clear precedent for surgical recordings. Additionally, this may facilitate conversation on storage and de-identification processes. While the hospital system may claim ownership and the right to prevent claims on derivative data, the hospital should establish an unequivocal delineation between ownership and patients’ rights. This measure would not only safeguard patients' rights by upholding their autonomy but also mitigate potential legal disputes that might arise.

No forms discussed legal uses for the recordings. This is pertinent for medicolegal considerations, as data collected for the sole purpose of quality improvement and surgical training may not be intended to be used in the patient’s medical record or used by patients’ legal representatives. In the instance of a serious adverse event resulting in legal proceedings [1, 34], a judge may decide to breach this protection and allow for the use of audiovisual data in the courtroom [18]. It is important to disclose this information in ICDs so that both patients and their medical teams understand the legal implications of audiovisual recording. More generally, legal and healthcare professionals may view ICDs as holding little weight in court. The utility and potential limitations of procedural audiovisual recordings in legal proceedings have been discussed for decades with no definitive consensus [18, 35–44]. Although ICDs can be legally binding in certain contexts such as organ donation [45], ICDs are often considered as framing instruments rather than binding contracts, documenting the patient’s agreement with the information provided but not always creating enforceable legal obligations [46]. Additionally, the ICD is intended to serve as a written record of patient understanding and agreement but does not reflect the nuance of concomitant in-person discussion. Despite the gap between verbal explanations and ICDs as a part of the entire consent process, the ICD constitutes a starting point for continued dialogue throughout an ongoing consent process.

### Limitations

A limitation of this study is that we had access only to ICDs and not any ancillary forms that hospitals may use. Our study examined the frequency of audiovisual recording disclosures in generic ICDs—forms intended for use across all procedures, regardless of whether recording is anticipated. While it is possible that some hospitals use separate forms for audiovisual recording that were unavailable to us, this does not account for the lack of discussion on patient rights and safeguards in nearly 86% and 50% of standard ICDs, respectively. Given that ICDs serve as the primary practical guide for physicians on which topics to cover during the consent process, the omission of these critical and increasingly relevant issues warrants concern. Inclusion of audiovisual recording disclosures—even when not universally applicable—would help ensure that patients are adequately informed. Although we may have missed additional topics or underestimated the prevalence of dedicated audiovisual consent forms, our sample of 70 ICDs yielded sufficient variability to detect rare but meaningful patterns in disclosure practices. Another limitation includes calculating the Flesch-Kincaid reading level since this might not be the optimal representation of readability; the amount of relevant text regarding intraoperative recording is often small compared to the text of the ICD in its entirety, which diminishes calculation efficacy. Regardless, most of the audiovisual disclosures we analyzed were clearly above the recommended 6th to 8th grade level. Additionally, the absence of a universally accepted gold standard makes it difficult to determine whether hospitals meet an appropriate standard of disclosure. The legal and ethical ambiguity surrounding data ownership and patient rights in non-identifiable surgical recordings may contribute to inconsistencies in disclosure, suggesting the need for further research to examine the underlying assumptions shaping institutional consent practices.

## Conclusion

Our analysis reveals that ICD language varies in its coverage of topics. Most but not all ICDs discussed video modality and the purpose of intraoperative recordings but typically failed to discuss safeguards and patient rights. The median reading grade level of audiovisual recording language was third year of college, exceeding recommended readability standards. Our findings help elucidate a clear tension between comprehensive coverage of important topics and maintaining appropriate readability; a high reading level is associated with inclusion of more subthemes, yet high ICD reading grade level likely hinders patient understanding. As more public attention is given to intraoperative recording, finding ways to discuss these critical topics in simplified, accessible language is necessary.

## Supplementary Information

Below is the link to the electronic supplementary material.

Supplementary file1 (DOCX 22 KB)

Supplementary file2 (DOCX 39 KB)
