# Supplementary material for: Patient rights and consent form language about intraoperative audiovisual recording
Source: Surg Endosc. 2025 Jul 17;39(9):5755–61. doi: 10.1007/s00464-025-12010-x (PMC12408661; doi:10.1007/s00464-025-12010-x)
Supplement: Supplementary file 2 — Supplementary file2 (DOCX 39 KB) [file 464_2025_12010_MOESM2_ESM.docx]

# Supplement 2. Data Analysis

# Aim 1: Explore the relationship between thoroughness and reading level of audiovisual consent language

## 1.0 Data

### 1.0.1 Hospital Demographics

| **Variable** | **Count (%)** |
| --- | --- |
| Census Region |  |
| Northeast | 10 (14%) |
| Midwest | 20 (29%) |
| South | 25 (36%) |
| West | 15 (21%) |
| Public | 29 (41%) |
| Academic | 30 (43%) |
| SVI, median (IQR) | 0.47 (0.33-0.67) |

SVI: Social Vulnerability Index; IQR: Interquartile range

### 1.0.2 Informed consent document characteristics

| **Characteristic** | **Median (IQR)** |
| --- | --- |
| Flesch-Kincaid Reading Level | 15.0 (12.5-17.7) |
| Characters | 184.5 (138.3-335.0) |
| Words | 35.5 (27.0-61.0) |
| Syllables per word | 1.8 (1.7-1.9) |

## 1.1 Statistical Models

### 1.1.1 Ordinal logistic regression: number of main themes and reading level

|  | **Odds Ratio** | **95% CI for Odds Ratio - Lower Bound** | **95% CI for Odds Ratio - Upper Bound** | **p-value** |
| --- | --- | --- | --- | --- |
| Region: Midwest | 0.162 | 0.033 | 0.793 | 0.028 |
| Region: South | 0.076 | 0.014 | 0.417 | 0.004 |
| Region: West | 0.149 | 0.028 | 0.787 | 0.029 |
| Public: Yes | 1.952 | 0.720 | 5.296 | 0.194 |
| Academic: Yes | 1.554 | 0.581 | 4.156 | 0.3836 |
| SVI | 31.455 | 2.670 | 370.512 | 0.0081 |
| Reading Level | 1.003 | 0.912 | 1.103 | 0.9482 |

As we can see from the table above, there is no evidence that reading level has an effect on number of main themes.

### 1.1.2 Ordinal logistic regression: number of subthemes and reading level

|  | **Odds Ratio** | **95% CI for Odds Ratio - Lower Bound** | **95% CI for Odds Ratio - Upper Bound** | **p-value** |
| --- | --- | --- | --- | --- |
| Region: Midwest | 0.287 | 0.075 | 1.096 | 0.0735 |
| Region: South | 0.332 | 0.083 | 1.327 | 0.1247 |
| Region: West | 0.268 | 0.062 | 1.154 | 0.0828 |
| Public: Yes | 1.293 | 0.532 | 3.140 | 0.5734 |
| Academic: Yes | 2.834 | 1.111 | 7.226 | 0.0336 |
| SVI | 1.620 | 0.202 | 12.997 | 0.6519 |
| Reading Level | 1.136 | 1.039 | 1.241 | 0.0069 |

As we can see from the table above, there is evidence that reading level has an effect on the number of subthemes (p < 0.01). As the odds ratio for reading level is greater than 1 (1.136), it indicates higher reading level leads to higher odds of having more subthemes.

# Aim 2 Part 1: Explore the relationship between inclusion of subthemes/number of themes and institution class

## 2.1 Statistical Models

### 2.1.1 Logistic regression: inclusion of research subtheme and institution class

|  | **Odds Ratio** | **95% CI for Odds Ratio - Lower Bound** | **95% CI for Odds Ratio - Upper Bound** | **p-value** |
| --- | --- | --- | --- | --- |
| Intercept | 0.039 | 0.005 | 0.316 | 0.0024 |
| Reading Level | 1.157 | 1.027 | 1.303 | 0.0164 |
| SVI | 5.002 | 0.451 | 55.475 | 0.1898 |
| Public: Yes | 0.527 | 0.177 | 1.571 | 0.2502 |
| Academic: Yes | 1.725 | 0.570 | 5.221 | 0.3343 |

As we can see from the table above, there is no evidence that institution class (public/not public, academic/not academic) has an effect on the inclusion of research subtheme.

### 2.1.2 Logistic regression: inclusion of observers subtheme and institution class

|  | Odds Ratio | 95% CI for Odds Ratio - Lower Bound | 95% CI for Odds Ratio - Upper Bound | p-value |
| --- | --- | --- | --- | --- |
| Intercept | 0.001 | 0.000 | 0.079 | 0.0015 |
| Reading Level | 1.206 | 1.017 | 1.430 | 0.0317 |
| SVI | 6.440 | 0.124 | 335.267 | 0.3557 |
| Public: Yes | 0.543 | 0.086 | 3.433 | 0.5163 |
| Academic: Yes | 2.181 | 0.372 | 12.781 | 0.3876 |

As we can see from the table above, there is no evidence that institution class (public/not public, academic/not academic) has an effect on the inclusion of observers subtheme.

### 2.1.3 Logistic regression: inclusion of education subtheme and institution class

|  | **Odds Ratio** | **95% CI for Odds Ratio - Lower Bound** | **95% CI for Odds Ratio - Upper Bound** | **p-value** |
| --- | --- | --- | --- | --- |
| Intercept | 0.112 | 0.011 | 1.123 | 0.0627 |
| Reading Level | 1.258 | 1.069 | 1.481 | 0.0057 |
| SVI | 0.476 | 0.028 | 8.079 | 0.6076 |
| Public: Yes | 1.560 | 0.428 | 5.685 | 0.5002 |
| Academic: Yes | 2.953 | 0.709 | 12.292 | 0.1367 |

As we can see from the table above, there is no evidence that institution class (public/not public, academic/not academic) has an effect on the inclusion of education subtheme.

### 2.1.4 Logistic regression: inclusion of deidentification subtheme and institution class

|  | **Odds Ratio** | **95% CI for Odds Ratio - Lower Bound** | **95% CI for Odds Ratio - Upper Bound** | **p-value** |
| --- | --- | --- | --- | --- |
| Intercept | 0.225 | 0.036 | 1.420 | 0.1126 |
| Reading Level | 0.951 | 0.856 | 1.057 | 0.3490 |
| SVI | 31.221 | 2.620 | 372.102 | 0.0065 |
| Public: Yes | 0.957 | 0.332 | 2.756 | 0.9352 |
| Academic: Yes | 1.670 | 0.579 | 4.814 | 0.3427 |

As we can see from the table above, there is no evidence that institution class (public/not public, academic/not academic) has an effect on the inclusion of deidentification subtheme.

### 2.1.5 Ordinal logistic regression: number of themes and institution class

|  | **Odds Ratio** | **95% CI for Odds Ratio - Lower Bound** | **95% CI for Odds Ratio - Upper Bound** | **p-value** |
| --- | --- | --- | --- | --- |
| Reading Level | 1.005 | 0.918 | 1.101 | 0.9062 |
| SVI | 15.600 | 1.728 | 140.849 | 0.0172 |
| Public: Yes | 1.333 | 0.530 | 3.354 | 0.5433 |
| Academic: Yes | 1.503 | 0.578 | 3.909 | 0.4062 |

As we can see from the table above, there is no evidence that institution class (public/not public, academic/not academic) has an effect on the number of main themes.

# Aim 2 Part 2: Explore the relationship between reading level and US region/SVI

## 3.1 Statistical Models

### 3.1.1 Linear regression: Reading level and US region, SVI

|  | **Estimate** | **95% CI for Estimate - Lower Bound** | **95% CI for Estimate - Upper Bound** | **p-value** |
| --- | --- | --- | --- | --- |
| Intercept | 12.363 | 8.288 | 16.437 | 0.0000 |
| Public: Yes | -0.275 | -2.762 | 2.212 | 0.8292 |
| Academic: Yes | -1.540 | -3.961 | 0.882 | 0.2172 |
| Region: Midwest | -1.014 | -4.822 | 2.795 | 0.6038 |
| Region: South | -0.330 | -4.202 | 3.542 | 0.8679 |
| Region: West | 2.173 | -1.861 | 6.207 | 0.2951 |
| SVI | 7.032 | 1.467 | 12.597 | 0.0160 |

As we can see from the table above, there is no evidence that US region has an effect on reading level. However, there is evidence that SVI has an effect on reading level. With every unit of increase in SVI, the reading level is expected to increase by 7.032.

# Software details

R version 4.4.1 (2024-06-14 ucrt)

Platform: x86_64-w64-mingw32/x64

Running under: Windows 11 x64 (build 22631)

Matrix products: default

locale:

[1] LC_COLLATE=English_United States.utf8

[2] LC_CTYPE=English_United States.utf8

[3] LC_MONETARY=English_United States.utf8

[4] LC_NUMERIC=C

[5] LC_TIME=English_United States.utf8

time zone: America/Chicago

tzcode source: internal

attached base packages:

[1] stats graphics grDevices utils datasets methods base

other attached packages:

[1] kableExtra_1.4.0 ggplot2_3.5.1 lmtest_0.9-40 zoo_1.8-12

[5] MASS_7.3-60.2 rms_6.8-0 expss_0.11.6 maditr_0.8.4

[9] table1_1.4.3 stringr_1.5.1 dplyr_1.1.4 Hmisc_5.1-2

loaded via a namespace (and not attached):

[1] gtable_0.3.5 xfun_0.43 htmlwidgets_1.6.4 lattice_0.22-6

[5] vctrs_0.6.5 tools_4.4.1 generics_0.1.3 sandwich_3.1-0

[9] tibble_3.2.1 fansi_1.0.6 highr_0.10 cluster_2.1.6

[13] pkgconfig_2.0.3 Matrix_1.7-0 data.table_1.15.4 checkmate_2.3.1

[17] lifecycle_1.0.4 compiler_4.4.1 MatrixModels_0.5-3 munsell_0.5.1

[21] codetools_0.2-20 SparseM_1.81 quantreg_5.97 htmltools_0.5.8.1

[25] yaml_2.3.8 htmlTable_2.4.2 Formula_1.2-5 pillar_1.9.0

[29] rpart_4.1.23 multcomp_1.4-25 nlme_3.1-164 tidyselect_1.2.1

[33] digest_0.6.35 polspline_1.1.24 mvtnorm_1.2-4 stringi_1.8.3

[37] splines_4.4.1 fastmap_1.1.1 grid_4.4.1 colorspace_2.1-0

[41] cli_3.6.2 magrittr_2.0.3 base64enc_0.1-3 survival_3.7-0

[45] utf8_1.2.4 TH.data_1.1-2 foreign_0.8-86 withr_3.0.0

[49] scales_1.3.0 backports_1.4.1 rmarkdown_2.26 matrixStats_1.3.0

[53] nnet_7.3-19 gridExtra_2.3 evaluate_0.23 knitr_1.46

[57] viridisLite_0.4.2 rlang_1.1.3 glue_1.7.0 xml2_1.3.6

[61] svglite_2.1.3 rstudioapi_0.16.0 jsonlite_1.8.8 R6_2.5.1

[65] systemfonts_1.0.6
